# Acute respiratory infections among individuals seeking outpatient care in the states of Washington and Michigan by pregnancy status, 2011–2016

**DOI:** 10.1111/irv.13230

**Published:** 2023-12-06

**Authors:** Collrane Frivold, Denise J. McCulloch, Seda Ekici, Emily T. Martin, Michael L. Jackson, Helen Y. Chu

**Affiliations:** ^1^ Department of Medicine University of Washington Seattle Washingtion USA; ^2^ Department of Epidemiology University of Washington Seattle Washingtion USA; ^3^ Department of Pediatrics University of Washington Seattle Washingtion USA; ^4^ Department of Epidemiology University of Michigan Ann Arbor Michigan USA; ^5^ EpiAssist LLC Seattle Washingtion USA

**Keywords:** infectious complications, influenza, maternal immunization, pregnancy, respiratory syncytial virus, respiratory viruses

## Abstract

**Background:**

Acute respiratory infections (ARIs) during pregnancy are associated with poor maternal and fetal outcomes.

**Methods:**

Using U.S. Flu Vaccine Effectiveness Network data (2011–2016) from Washington and Michigan, we tested for respiratory viruses among pregnant and non‐pregnant outpatients matched on age, site, and season (*n* = 191).

**Results:**

Among all participants, detection of human coronaviruses and rhinovirus was common. We also observed differences in virus detection by pregnancy status; human coronaviruses and respiratory syncytial virus (RSV) were detected more frequently among pregnant and non‐pregnant participants, respectively.

**Conclusions:**

The role of respiratory viruses in maternal ARI morbidity should be further characterized to inform implementation of prevention interventions including maternal vaccines.

## 1 BACKGROUND

Respiratory infections are considered common during pregnancy.^1^, ^2^ For instance, a cross‐sectional study of pregnant outpatients in the United States identified acute lower respiratory tract illness among 36% of pregnant participants with acute respiratory illness (ARI) symptoms (29/81).^1^ Pregnancy is also a potential risk factor for adverse outcomes following ARI including increased maternal morbidity/mortality^3^, ^4^, ^5^, ^6^ and adverse perinatal outcomes,^3^, ^5^, ^6^, ^7^ which have been reported for both influenza and SARS‐CoV‐2 infections.

Although maternal influenza and SARS‐CoV‐2 infections have been previously studied and vaccines against these pathogens are available for use in pregnancy to prevent adverse maternal and fetal/infant outcomes, other common respiratory pathogens such as respiratory syncytial virus (RSV),^8^ human coronaviruses, and rhinovirus remain understudied among pregnant populations at the population level in spite of rapid development of vaccines intended for use during pregnancy.

Our primary objective was to compare respiratory virus detection, illness characteristics, and subsequent hospitalizations among pregnant and non‐pregnant individuals of reproductive age with ARI symptoms in the United States who were seeking care in an outpatient setting at enrollment. As new vaccines against respiratory viruses indicated for use in pregnancy progress towards licensure and subsequent introduction, such as Pfizer's maternal RSV vaccine approved in August 2023,^9^, ^10^ additional studies are needed to further characterize the maternal burden of respiratory viruses across multiple years and to inform future implementation of these prevention products.

## 2 METHODS

### 2.1 Data source

The Centers for Disease Control and Prevention (CDC) U.S. Flu Vaccine Effectiveness (VE) Network conducts active surveillance of influenza vaccine effectiveness in preventing medically attended ARI (MAARI) using a test‐negative design.^11^, ^12^ Individuals presenting for care at participating health‐care facilities are invited to participate if they meet a standard illness case definition (i.e., fever/cough or cough alone) and are at least 6 months of age. We used data from the Michigan and Washington U.S. Flu VE Network sites collected between the 2011/2012–2016/2017 respiratory virus seasons for participants ≥18 years of age. During the 2011/2012 season, ARI was defined as a respiratory illness with fever or cough. In subsequent seasons (2012/2013–2016/2017), ARI was defined as a cough with ≤7 days duration. Institutional Review Board (IRB) approval for the Flu VE Network protocol was granted to all participating sites. This study was approved by the Kaiser Permanente Washington IRB.

### 2.2 Data collection

All eligible and consenting U.S. Flu VE Network participants completed an enrollment interview and provided combined nasal and oropharyngeal swabs for respiratory virus testing in the investigators' laboratories. Influenza A/B testing was performed via reverse transcription polymerase chain reaction (RT‐PCR) using CDC‐provided primers and probes as previously published.^11^, ^12^ The Washington and Michigan sites tested for the same non‐influenza viruses: Seasonal human coronaviruses [229, 43, 63, HKU], rhinovirus, human metapneumovirus, RSV, adenovirus, bocavirus, parainfluenza 1–4, enterovirus, and parechovirus. The Washington site used RT‐PCR as previously described^13^; the Michigan site used multiplex primers and probes obtained from Fast‐Track Diagnostics (Siemens Healthineers, Germany). Since these data were collected prior to the COVID‐19 pandemic, we did not test for SARS‐CoV‐2.

Additional data on sociodemographic factors, illness characteristics, disease severity, and illness outcomes were obtained through medical record extraction. Influenza vaccination status was confirmed through electronic medical records and state immunization registries.

### 2.3 Study population

We included female adults (≥18 years of age) enrolled in the U.S. Flu VE Network within the Washington and Michigan sites. Participants were recruited from Kaiser Permanente Washington (formerly Group Health Cooperative, Seattle, Washington), ambulatory clinics within Michigan Medicine (Ann Arbor, Michigan), and the Henry Ford Health (Detroit, Michigan).

### 2.4 Exposure

The exposure of interest was self‐reported pregnancy status.

### 2.5 Outcomes

The primary outcome was detection of a respiratory virus (yes/no) using RT‐PCR. A positive test result (i.e., virus detected) was defined separately for each virus. As a secondary outcome, we evaluated whether a participant was hospitalized for a MAARI following enrollment based on the presence of an ICD‐10 MAARI diagnosis code (yes/no).

### 2.6 Covariates

Pregnant and non‐pregnant participants were matched 1:1 on age in years, site (Washington or Michigan), and the respiratory virus season at enrollment (2011/2012–2016/2017).

### 2.7 Statistical analyses

We first conducted descriptive analyses on demographics, illness characteristics, and respiratory virus detection. We calculated the number and proportion of illness episodes where a respiratory virus was detected. Using conditional logistic regression, we calculated the odds ratio (OR) and associated 95% confidence interval (CI) of hospitalization following the ARI diagnosis by pregnancy status at enrollment. Study participants were analyzed as matched sets conditioning on age, site, and respiratory virus season. Although we performed the RT‐PCR assay on all matched sets, we excluded participants if RT‐PCR results could not be linked to the self‐reported pregnancy status variable included in the analytic dataset (*n* = 5). This resulted in unequal numbers of pregnant/non‐pregnant participants after matching. All statistical analyses were performed using R version 4.0.2 (R Foundation for Statistical Computing) in RStudio Version 3.1073 (RStudio, Inc; Boston, MA).^14^

## 3 RESULTS

A total of 191 individuals met the inclusion criteria including 93 pregnant and 98 non‐pregnant individuals (Table 1). Participation by year was as follows: *n* = 26 in 2011/2012, *n* = 16 in 2012/2013, *n* = 37 in 2013/2014, *n* = 42 in 2014/2015, *n* = 52 in 2015/2016, and *n* = 18 in 2016/2017. Baseline characteristics were similar across pregnancy exposure groups. Key differences were that fewer pregnant participants identified as black (6.5% versus 16.3%), Hispanic (3.2% versus 6.1%), and were current smokers (4.3% versus 10.2%). A larger proportion of pregnant participants had also received an influenza vaccine in the past year (61.3% versus 35.7%).

**TABLE 1:** Baseline characteristics by pregnancy status (*n* = 191).

Baseline characteristics	Pregnant (%) (*n* = 93)	Non‐pregnant (%) (*n* = 98)
Site		
Washington	55 (59.1%)	60 (61.2%)
Michigan	38 (40.9%)	38 (38.8%)
Race^a^		
White	74 (79.6%)	68 (69.4%)
Black	6 (6.5%)	16 (16.3%)
Asian	7 (7.5%)	9 (9.2%)
Native Hawaiian	1 (1.1%)	3 (3.1%)
American Indian	1 (1.1%)	1 (1.0%)
Other	4 (4.3%)	4 (4.1%)
Refused	1 (1.1%)	3 (3.1%)
*Missing*	*4 (4.3%)*	*4 (4.1%)*
Hispanic	3 (3.2%)	6 (6.1%)
*Missing*	*0 (0%)*	*1 (1.0%)*
Education		
	2 (2.2%)	1 (1.0%)
High school	8 (8.6%)	6 (6.1%)
Some college	23 (24.7%)	29 (29.6%)
Bachelor's degree	20 (21.5%)	21 (21.4%)
Advanced degree	19 (20.4%)	19 (19.4%)
Refused	1 (1.1%)	0 (0%)
*Missing*	*20 (21.5%)*	*22 (22.4%)*
Current smoking		
Yes	4 (4.3%)	10 (10.2%)
No	89 (95.7%)	86 (87.8%)
Do not know/refused	0 (0%)	2 (2.0%)
Asthma	13 (14.0%)	15 (15.3%)
*Missing*	*18 (19.4%)*	*21 (21.4%)*
Cardiac comorbidities	2 (2.2%)	3 (3.1%)
Diabetes	3 (3.2%)	3 (3.1%)
Received influenza vaccine	57 (61.3%)	35 (35.7%)

^a^ Race categories are not mutually exclusive.

**FIGURE 1:**
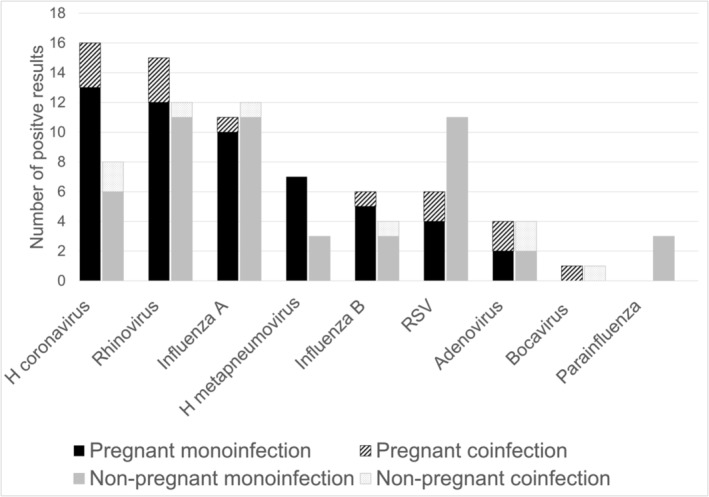
Detection of respiratory viruses via RT‐PCR by pregnancy status 2011–2016 (*n* = 191).^1,2^ The number of times a single virus was detected (monoinfection) is represented by solid shading; the detection of more than one virus (coinfection) is represented by diagonal line shading. Abbreviations: H. coronavirus, human coronavirus; H. metapneumovirus, human metapneumovirus; RSV, respiratory syncytial virus; RT‐PCR, reverse transcription polymerase chain reaction. ^1^No enterovirus or parechovirus detected. ^2^Ten total coinfections with two (*n* = 9) or three (*n* = 1) viruses.

At least one virus was detected in 59.2% of the participants (*n* = 113) including 63.4% pregnant (*n* = 59) and 55.1% non‐pregnant participants (*n* = 54). Both respiratory virus monoinfections (detection of one virus) and coinfections (detection of multiple viruses) were identified with monoinfections being more common (Figure 1). A total of 10 coinfections were detected with two (*n* = 9) or three (*n* = 1) viruses. Among pregnant participants, the most common pathogens detected were human coronaviruses (*n* = 16), rhinovirus (*n* = 15), and influenza A (*n* = 11). Among non‐pregnant participants, the most common pathogens detected were rhinovirus (*n* = 12), influenza A (*n* = 12), and RSV (*n* = 11). When comparing the number of positive PCR results among pregnant and non‐pregnant participants for a given virus, the greatest difference in the number of positive PCR specimens was observed for human coronavirus (*n* = 16 pregnant; *n* = 8 non‐pregnant), human metapneumovirus (*n* = 7 pregnant; *n* = 3 non‐pregnant), RSV (*n* = 6 pregnant; *n* = 11 non‐pregnant), and parainfluenza (*n* = 0 pregnant; *n* = 3 non‐pregnant).

Regarding illness characteristics, 9.7% of pregnant participants (9/93) compared to 2.0% of non‐pregnant participants (2/98) were hospitalized for a MAARI following enrollment. In this study population, the estimated odds of hospitalization for a MAARI following enrollment among pregnant participants was 4.0 times that of non‐pregnant participants (95% CI: 0.5–35.8).

## 4 DISCUSSION

We detected a wide range of respiratory viruses, which differed by pregnancy status. For instance, human coronaviruses were detected more frequently among pregnant participants and RSV was detected more frequently among non‐pregnant participants. Viral detection was also more common among pregnant participants (63.4% vs 55.1%). Although a greater proportion of pregnant participants had received a seasonal influenza vaccine (61.3% vs 35.7%), a similar proportion of influenza A/B infections were detected (18.5% vs 16.7%).

In this study population, we estimated that the odds of hospitalization for a MAARI following enrollment were also higher among pregnant participants. However, the association of interest was not statistically significant. The wide 95% CI may be because of the small sample size. This observed association could be related to several factors such as ARI disease severity or clinicians being more likely to admit pregnant participants to facilitate monitoring.

### 4.1 Strengths and limitations

A key strength of this analysis is that we performed testing for 10 respiratory viruses including subtyping across multiple seasons and distinguished between whether a single virus or multiple viruses were detected. Furthermore, study participants represented two U.S.‐based geographically distinct sites.

Matching on age, study site, and respiratory virus season also enabled our exposure groups to be more exchangeable. However, the generalizability of our results may be limited because the Flu VE Network only recruits from outpatient settings. Therefore, individuals who do not seek care at these locations are excluded, which could have impacted our findings. Furthermore, the Flu VE Network does not recruit from obstetrics/gynecology clinics where pregnant individuals may seek care for an ARI as part of their prenatal care. Another potential limitation is that these data were collected prior to the onset of the COVID‐19 pandemic. Therefore, we are not able to comment on how detection of SARS‐CoV‐2 compares to other viruses in this study population.

### 4.2 Future directions

To overcome sample size limitations of this analysis, larger studies of nationally representative pregnant populations are needed to fully characterize the maternal ARI burden. To improve generalizability, future studies should also be conducted among diverse pregnant populations at different types of health facilities to represent a range of health‐care‐seeking behaviors or at the community level among those not seeking care. Generating these data are critical to inform the implementation of disease prevention interventions targeted at pregnant populations including maternal RSV vaccines currently in the pipeline. These maternal ARI burden data will be critical to understand how the introduction of new prevention interventions may impact the burden of respiratory infections during pregnancy.

## AUTHOR CONTRIBUTIONS

Collrane Frivold performed the final analysis and wrote the first draft of the manuscript. Helen Y. Chu, Michael L. Jackson, and Emily T. Martin contributed to the conceptualization of the research question and study design as well as reviewed and contributed to the writing of the manuscript. Seda Ekici and Denise J. McCulloch performed a preliminary analysis as well as reviewed and contributed to the writing of the manuscript. All co‐authors reviewed and approved the article prior to submission.

## CONFLICT OF INTEREST STATEMENT

HYC reported consulting with Ellume, Pfizer, The Bill and Melinda Gates Foundation, Glaxo Smith Kline, and Merck. She has received research funding from Emergent Ventures, Gates Ventures, Sanofi Pasteur, the Bill and Melinda Gates Foundation, and support and reagents from Ellume and Cepheid outside of the submitted work. ETM reported research funding from Merck outside of the submitted work. CF, MLJ, SE, and DJM reported no competing financial interests.

### PEER REVIEW

The peer review history for this article is available at https://www.webofscience.com/api/gateway/wos/peer-review/10.1111/irv.13230.

## ETHICS APPROVAL STATEMENT

Institutional Review Board approval for the Flu VE Network protocol (parent study) was granted to all participating sites. This study was approved by the Kaiser Permanente Washington IRB.

## PATIENT CONSENT STATEMENT

Patients were eligible to participate if they presented for medical care at health‐care facilities participating in the Flu VE Network, met a standard illness case definition (i.e., fever/cough or cough alone), and were 6 months of age and older. All participants or their guardians provided informed consent prior to participation in the Flu VE Network.

## Data Availability

Data not publicly available.
